# Assessing Elderly User Preference for Telehealth Solutions in China: Exploratory Quantitative Study

**DOI:** 10.2196/27272

**Published:** 2022-01-12

**Authors:** Nuoya Chen, Pengqi Liu

**Affiliations:** 1 Faculty of Global Studies, Justice and Rights University of Macerata Macerata Italy; 2 Sino-Danish College University of Chinese Academy of Sciences Beijing China

**Keywords:** telehealth solutions, preference, motivation, elderly user, China

## Abstract

**Background:**

In the next 15 to 20 years, the Chinese population will reach a plateau and start to decline. With the changing family structure and rushed urbanization policies, there will be greater demand for high-quality medical resources at urban centers and home-based elderly care driven by telehealth solutions. This paper describes an exploratory study regarding elderly users’ preference for telehealth solutions in the next 5 to 10 years in 4 cities, Shenzhen, Hangzhou, Wuhan, and Yichang.

**Objective:**

The goal is to analyze why users choose telehealth solutions over traditional health solutions based on a questionnaire study involving 4 age groups (50-60, 61-70, 71-80, and 80+) in 4 cities (Shenzhen, Hangzhou, Wuhan, and Yichang) in the next 10 to 20 years. The legal retirement age for female workers in China is 50 to 55 years and 60 years for male workers. To simulate reality in terms of elderly care in China, the authors use the Chinese definition of elderly for employees, defined as being 50 to 60 years old rather than 65 years, as defined by the World Health Organization.

**Methods:**

The questionnaires were collected from Shenzhen, Hangzhou, Wuhan, and Yichang randomly with 390 valid data samples. The questionnaire consists of 31 questions distributed offline on tablet devices by local investigators. Subsequently, Stata 16.0 and SPSS 24.0 were used to analyze the data. O-logit ordered regression and principal component analysis (PCA) were the main theoretical models used. The study is currently in the exploratory stage and therefore does not seek generalization of the results.

**Results:**

Approximately 71.09% (280/390) of the respondents reported having at least 1 type of chronic disease. We started with PCA and categorized all Likert scale variables into 3 factors. The influence of demographic variables on Factors 1, 2, and 3 was verified using analysis of variance (ANOVA) and *t* tests. The ordered logit regression results suggest that health-related motivations are positively related to the willingness to use telehealth solutions, and trust on data collected from telehealth solutions is negatively correlated with the willingness to use telehealth solutions.

**Conclusions:**

The findings suggest that there is a need to address the gap in community health care and ensure health care continuity between different levels of health care institutions in China by providing telehealth solutions. Meanwhile, telehealth solution providers must focus on improving users’ health awareness and lower health risk for chronic diseases by addressing lifestyle changes such as regular exercise and social activity. The interoperability between the electronic health record system and telehealth solutions remains a hurdle for telehealth solutions to add value in health care. The hurdle is that doctors neither adjust health care plans nor diagnose based on data collected by telehealth solutions.

## Introduction

### Background

COVID-19 has severe effects on the elderly population with multiple chronic diseases such as hypertension, diabetes, and cardiovascular diseases than the healthy subpopulation. Mortality rate analysis shows higher death rates caused by COVID-19 associated with those aged above 50 years [1]. Population projections suggest that the Chinese population will peak from 2025 to 2030 [2], thereby leading to surging demands for high-quality medical services including telehealth solutions. Research on the preference of the elderly for telehealth solutions must be conducted. The legal retirement ages for female and male employees in China are 55 and 60 years respectively, ranking as one of the lowest in the world [3]. Therefore, to study elderly user preferences, the authors chose to start from those aged 50 years to simulate reality at the best possible level in this study.

Telehealth refers to the use of telecommunication tools for health care continuum in the prevention, treatment, diagnosis, recovery, and home care processes. The use of wearables and apps for health management and online hospitals for health consultation has become increasingly popular with the wide use of smartphones. COVID-19 has accelerated the digitalization of the health care system at a pace unimaginable a few years ago. The use of telemedicine services has increased by more than 1000% in March and more than 4000% in April [4]. Spending on the use of telehealth solutions also increased starting from March by more than 1000 % [5].

There is a need to explore user preference for telehealth solutions, particularly for the future generations of elderly (aged 50 years and above) who will become 70 years old by 2030. The Chinese society is facing challenges posed by a rapidly aging population and rising chronic disease trends caused by lifestyle changes owing to the urbanization process. Based on projections, the Chinese population will peak between 2026 and 2030 [2]. Thus, researching whether and how telehealth solutions can generate more value for the elderly in the next 15 to 20 years is important. Owing to the lack of high-quality medical resources and trained clinicians, there is an urgent need to look for alternative solutions such as telehealth solutions. The implementation of telehealth solutions faces challenges among elderly users given their lack of experience with technology and thereby the lack of trust in telehealth solutions. Other factors such as the household income, education level, and health status of the users may also play a role.

The rest of the paper is structured as follows. The next section provides the literature review on research methodologies used to study users’ willingness for using telehealth solutions. We then present the methodology and research design used for the analysis, followed by the description of the qualitative and quantitative analyses of the questionnaire revealing why users choose telehealth solutions over traditional health solutions. Finally, we summarize the main findings and implications of this study.

### Literature Review

To analyze the state of the art of the research methodology regarding user and physician preferences for telehealth solutions, a thorough literature review was conducted.

There have been several empirical studies on patients in all age groups and clinician perceptions regarding telehealth solutions. The multinominal logit regression model has become popular for statistical analyses in health economics and marketing science [6]. The paired *t* test has also been also used for comparing the preference for traditional health visits with telehealth consultations or the presence of telephysicians.

Direct-to-consumer telehealth solutions roughly comprise 3 categories [6]. The first category covers solutions provided by the same doctor from whom the patients obtain primary care services. As the health care service is provided by the doctor with whom patients have established a relationship, telehealth solutions can ensure convenience to patients while maintaining care continuity. The second category incorporates solutions provided by doctors from the same institution where patients receive health care services but not the same doctor with whom the patients have an established relationship. This allows the patients’ records to be updated by the doctor from the same care institution while maintaining the connection with the care home. Meanwhile, patients can receive care during and after working hours. The third category consists of telehealth solutions provided by doctors who have no previous relationship with the patients or the patients’ primary care service providers. Many newly emerging telehealth solution providers belong to the third category. Patients can pay for the services provided out of their pockets or by claiming insurance.

One study [7] used SurveyMonkey to send out a questionnaire for conducting a nation-wide survey in the United States. In total, there were 4345 patients covering different ethnicities, age groups, and income groups with various education backgrounds and insurance coverages. The survey aimed to determine the willingness of the participants to use telehealth solutions and their comfort level with telehealth solutions belonging to the aforementioned 3 categories of solutions. Results from the generalized estimation equation model showed that patients were more willing to use category I solutions. The willingness to use telehealth solutions declined if the provider had no relationship with patients before or if the services were provided by other doctors from the same care institutions. More than half of the patients were willing or very willing to use telehealth solutions involving their own doctor. One-third of all participants were willing to use telehealth solutions provided by other doctors from the same care institution. Less than 20% of all participants were willing to use telehealth solutions provided by doctors with whom they had no previous relationship. Patients’ comfort in using telehealth solutions grows with the attachment to their original care institution.

In another study [8], the authors have tried to analyze patient preferences and satisfaction rates with the telehealth program, CVS Minute Clinics. Minute Clinics offer patients video consultation with doctors at collaboration clinics while assisting nurses in performing on-site diagnostic tests and using tools such as otoscopes, telephonic stethoscopes, and digital video laryngoscopes to assist doctors in making diagnoses by reading the image or data on the screen. Such treatment costs US $59 on average with life insurance [9].

The survey participants were over 18 years old and agreed to use telemedicine service when on-site doctors were busy. The study used the logistic regression model to assess the preferences of 1734 users of the Minute Clinics services. Among these participants, 94% to 99% reported high satisfaction with telehealth solutions. One-third of all participants preferred telehealth solutions to traditional health solutions. The authors suggest that the lack of medical insurance, gender (female) of the users, self-satisfaction with the understanding of telehealth solutions, service quality, and convenience can predict user preference for telehealth visits [10]. Patients’ satisfaction with on-site nurses has an adverse relationship with the preference for telehealth solutions. The possible explanations are that the more satisfied patients are with on-site nurses, the more they are reminded of the benefits of in-person interactions. Moreover, patients may get the false impression that on-site nurses alone can perform the necessary diagnosis and therefore ignore the fact that on-site nurses do not have the license to practice alone.

One study [11] has analyzed factors associated with clinicians’ perceptions regarding telehealth solutions and examined if these factors affected their decision to continue using telehealth solutions after COVID-19. Doctors from different disciplines, including pediatricians and doctors focusing on adult patients, surgical and nonsurgical doctors, outpatient and inpatient doctors, and doctors who focus on both categories have been covered [10]. The 220 full responses also covered doctors with and without previous telehealth experiences. The study disseminated a Likert scale questionnaire and used logistic regression to analyze the odds of different factors at a significance level of 95%.

Results [10] suggest that ease of use for patients is the most important feature followed by ease of use for clinicians. Physicians’ overall satisfaction [11] and perceived ease of use [12] also directly affect perceived usefulness and the intention to use telemedicine. Meanwhile, the quality of care, ease of physical examination, and beliefs on whether adaptability is an important quality of clinicians also play a role in determining doctors’ preference for telehealth solutions. Being more perceiving rather than judging is also seen as one of the personality factors affecting clinicians’ decision to extend their use of telehealth solutions. Moreover, clinicians’ beliefs regarding the importance of physical touch have a negative correlation with their decision to extend the use of telehealth solutions.

The study conducted by Miner [10] suggests that clinicians play a significant role in adapting to the digital health trends. Training may prove necessary to help clinicians continue their telehealth practices after COVID-19.

Researchers [13] have studied outpatients’ use of the internet to search for orthopedic information. They used a questionnaire consisting of 12 questions that was distributed by doctors to outpatients during office visits. A total of 1161 complete responses were collected and analyzed with a multivariable binominal logistic regression model. Regression results show that younger age groups are primarily associated with increased use of the internet for obtaining health and orthopedic information. Younger patients are also more likely to find the search results related to their current orthopedic problems “very helpful” and “somewhat helpful.” Google is a more popular search engine than Yahoo and Bing. Patients who visited sports medicine clinics were less likely to use WebMD to search for answers to their orthopedic questions. Other than this, the type of clinic did not have a significant effect on patients’ use of the internet. Males were more likely to find information from the internet very useful than female patients; besides this, gender does not have a significant impact on patients’ internet usage. The study suggests that patients seem to conduct research on the internet with search engines more than on the website of the institution where they are being treated.

Another study [14] confirms that using the internet for searching information along with telehealth solutions, and doctors’ suggestions in clinics and hospitals shall address the problem where patients rely on search engines to search answers to medical problems because of the lack of reliable medical information sources online. Chatbots can offer an alternative for such a problem.

The study [14] compared the accuracy of traditional nurse triages and physician telepresence at an emergency pediatric department. The study used paired *t* tests to analyze the triage time and accuracy (triage utility) differences between traditional nurse triages and physician telepresence. In total, data on 100 families were collected in this study, which took place at a large, tertiary care children’s hospital with 65,000 emergency department visits occurring annually. Physician telepresence was achieved using the RP-7i robot, with a built-in stethoscope after the patients went through the traditional nurse triage. The questionnaire consists of 9 5-point Likert scale questions and 1 yes/no question to assess the overall experience of using the robot.

At *P*=.10, there is no difference in the triage time between the traditional nurse triage and physician telepresence. There are statistically significant differences between the triage accuracy of traditional nurse triages and physician telepresence (*P*=.03). The triage accuracy score of the traditional nurse triage is at 71% whereas the physician telepresence score is at 95%. Parents and children have preference scores for physician telepresence and indicate that they would choose physician telepresence during their next pediatric emergency department visit [14].

Another study [15] focused on children who were 5.99 years old on average. These children preferred new technology. In the emergency department, time is everything whereas it may be tricky for nurses to make accurate judgments without enough physicians in the emergency room. The robotic experience has significantly improved triage accuracy by avoiding missing values on the triage form, which consists of 27 items. This suggests that in an overwhelmed emergency room, having physician telepresence may help ease stress and avoid mistakes.

The study [15] also analyzed the impact of the integrated health care buddy project with patients having chronic disease conditions in the United States. The study is a collaboration study between 2 clinics at Washington and Oregon, Robert Bosch Healthcare and American with 2 groups of patients (an intervention group and a control group), each comprising 1767 patients with chronic obstructive pulmonary disease, congestive heart failure, or diabetes. The health buddy program gives a free handheld device for patients to use at home and a large screen. The device connects patients with care managers and allows patients to interact with their care managers about vital signs, symptoms, health-related knowledge, and behavior. Insurance claim data were used to analyze the cost for managing chronic disease and mortality rates.

Moreover, the study confirms the effectiveness of harnessing telehealth assistants for chronically ill patients. Telehealth solutions not only lowered the mortality rates by 2.7% in the intervention group over 2 years but also saved costs between 7.7% and 13.3% per patient per quarter (US $312-542). The study used multivariate regression to predict the cost reduction for patients who engaged more with the program and patients who do not engage otherwise. The prediction showed cost savings of US $1009 per congestive heart failure patient per quarter (*P*<.001). For patients engaged in the program, the cost saving is US $968 per patient per quarter (*P*<.001). For patients who did not engage with the program, the cost saving is not significant. For hospital admission rates, the study suggests that telehealth intervention lowers inpatient admission by 3.4% (*P*<.001).

The paper highlights the need to recognize the value of integrated telehealth solutions for high-risk patients with chronic diseases who incur high costs. Having a device at home for allowing patients to interact with care managers not only allows care managers to capture the deteriorating vital signs and provide interventions in time but also to identify gaps in patients’ health knowledge and behavior [15].

The discrete choice experiment (DCE) has been a popular tool to identify the preference for telehealth solutions and the different attributes related to the preference for such solutions [16]. Researchers tried to identify the preference of elderly people (aged 65 years and above) in Australia. The study [16] analyzed factors such as the distance to the nearest clinic and cost of virtual visits and their influence on the preference level for telehealth solutions. The study indicates that most of the elderly have never used the internet in the past 3 months, indicating a knowledge gap for elderly users in using telehealth solutions. In the study, 330 respondents were recruited with a mean age of 69 years. The study concludes that participants would rather use telehealth solutions only as complementary tools with in-person visits. As the study was conducted in Adelaide, Australia, where the age structure, family structure, and health status of the elderly are different from those in China, there is a need to analyze the preference of elderly users for telehealth in China.

In another study [17], the choice between mobile health and telehealth was studied with the DCE model involving 1403 residents in rural areas. The study suggests that the preference is associated with the gender and setting of the users. The distance (access to health care) to hospitals and their gender determines if the residents would prefer using telehealth solutions.

## Methods

### Questionnaire Distribution

With the legal retirement age standing at 55 years for female employees and 60 years for male employees in China, the questionnaire was distributed among the future elderly (aged above 50 years) in Shenzhen, Hangzhou, Wuhan, and Yichang to best simulate reality. This study followed the DCE methodology and comprised 5 stages including designing research questions, interviews with experts, interviews with individual users, the pretest stage, and the pilot test stage. The questionnaire study was conducted with assistance from the University of Chinese Academy of Sciences and Beijing Cinsos Consulting Corporation. We collected 390 valid answers from 50-60, 60-70, 70-80, and 80+ age groups to analyze individual users’ willingness to use telehealth solutions over traditional health solutions.

### Ethical Approval

Ethical approval was obtained in May 2019 from the committee in University of Macerata.

Based on the ethical approval results and the analytical results from focus group analysis, questionnaires were designed to analyze stakeholders’ attitudes in China toward whether the Internet of Healthcare Things solutions can help reduce the gap in the demands of the current health care system.

Table 1 summarizes the collected data. All data were stored on the “Box” owned by KU Leuven.

**Table 1:** Data collection summary (source: author’s design).

Data	Format	Transfer	Consent	Pseudonymization
Recording with consumers	Windows Media Audio (WMA), MP3, MP4	Data were collected for scientific research purposes and therefore transferred from China to Europe and stored on cloud.	Question 1 in the questionnaire (see Multimedia Appendix 1)	Yes
Questionnaire collected on tablet devices	Word	Data were collected for scientific research purposes and therefore transferred from China to Europe and stored on cloud.	Question 1 in the questionnaire (see Multimedia Appendix 1)	Yes
Excel form with summary of data pseudonymized	Excel	Data were collected for scientific research purposes and therefore transferred from China to Europe and stored on cloud.	Question 1 in the questionnaire (see Multimedia Appendix 1)	Yes

### Study Design

The main purpose of the survey was to understand the factors affecting the preference of elderly users for telehealth solutions. The DCE model based on the random utility theory to evaluate the preference for telehealth solutions [18] was used. The DCE method is widely used in studying how patients value different attributes of health care services and the potential demand for new services or treatment [19]. The study follows the standard DCE methodology, namely (1) defining research questions to compile evidence, (2) interviewing experts (stakeholders), (3) interviewing individual users(focus group studies), (4) the pretest stage (online questionnaire in Europe, N=31), and (5) the pilot test stage (online questionnaire in Xiangyang, China, N=104). In the pilot test stage, 104 questionnaires were answered, with 55 questionnaires containing usable data (mostly from Hubei Province).

The questionnaire (see Multimedia Appendix 1) consists of 31 questions and 5 parts. The questionnaire starts with a screening question on whether the participant is willing to participate in the survey and share data for scientific research purposes. There are 10 Likert scale questions related to the motivation, 7 questions surrounding the demographic information including participants’ insurance coverage, 6 questions about the usage of telehealth solutions at the time of survey, 4 questions about the health status of survey participants, and 3 questions about whether users want to share data with insurance companies, doctors from community health centers, and doctors from hospitals. The degree of influence of each factor is evaluated with a Likert scale from 1 to 7 (1=no influence, 4=neutral, and 7=with influence). The questionnaire was written in Chinese and then translated in English for easy understanding by the author.

The questionnaire has 5 parts; the first part is about the current situation of telehealth solution usage by the surveyed elderly users.

Telehealth solutions are defined as smartphone apps (such as Alihealth, Ping An Good Doctor, Chun Yu Doctor, Wedoctor, Yue Dong Quan, etc), wearables (such as Xiaomi Band, Huawei watch and Apple Watch, etc), health management tools for home use (such as PICOOC smart scale, Mi Home i-Health blood pressure monitor, Mi Home Hi-Pee Smart Pee Monitor, Smart Sleep Monitor, Smart devices to improve sleep quality, etc.). The section consists of 4 questions asking the usage frequency, reasons for starting to use telehealth solutions, if telehealth solutions were used to monitor sleep, and if telehealth solutions were used to monitor nutrition.

The second part of the questionnaire is about the health status of the survey participants (self-evaluated). The third part asks about the potential benefits of telehealth solutions and elderly users’ motivations. The fourth part is designed around the potential risks of telehealth solutions (price, privacy risk, data accuracy risk, brand and design, resistance to technology, and usage experience). The fifth part is designed to gather demographic information, including gender, age, residence, household income, and education in years.

Further, 13 questions were designed focusing on the reasons for preferring telehealth solutions to traditional health solutions. The following questions are related to F2, the perceived benefits of telehealth solutions: monitoring health status (Q13), reducing health risks (Q14), following the doctor's advice (Q15), free devices provided by insurance companies (Q18), and lack of community health care services (Q20).

There were also questions regarding the perceived risk for telehealth solutions (F3), including data accuracy (trust) concerns (Q22), privacy concerns (Q23), financial reasons for the price (Q24), design, popularity, and usage difficulty concerns (Q25).

As some of the reasons for using telehealth solutions pertain to the social image of the individuals, social influence (Q28) is also considered one of the factors that could influence users’ preference.

### Data Collection

In China, the legal retirement age for female factory workers is 50 years, 55 years for female employees, and 60 years for male employees. To simulate reality with respect to elderly care in China, elderly is defined as being over 50 years old instead of being 65 years old according to the World Health Organization. In our study, we intended to compare the participants’ willingness to use telehealth solutions considering different age groups and residents in different cities, with the data collection target set for each age group (50-60, 61-70, and 71-80 years) containing approximately 100 data subjects. Data subjects more than 80 years old were categorized as being in the 50-100 years group because of the health conditions that limited the number of participants.

In the pretest stage of the study, questionnaires in English were distributed on the internet via Microsoft Forms through the Philips intranet portal and Berlin Expat Group on Facebook. We collected 31 questionnaires. In the pilot testing stage, the questionnaire was translated into Chinese and distributed via the internet with Wenjuanxing through WeChat. We collected 104 questionnaires with 55 valid answers. The pretesting stage was designed to test the design of the questionnaire; therefore, the data collected were not analyzed.

In the distribution stage, questionnaires were disseminated on tablet devices by local investigators randomly among residents more than 50 years old in Shenzhen, Hangzhou, Wuhan, and Yichang with the help of Beijing Cinso Consulting Corporation. More than 450 questionnaires were distributed, and 402 answers were collected, with a recovery rate of 89%. Among them, 390 were completely valid questionnaires, accounting for 87% of the questionnaires issued and 97% of all the questionnaires returned. The other 12 questionnaires were not used in data analysis because they did not provide complete information or were deemed to have not been filled carefully.

The data were collected in Chinese language and then summarized in an Excel sheet (Microsoft Corporation) and converted into a pseudonymized value form in Excel. Data were then analyzed using SPSS 24.0 (IBM Corporation) and Stata 16.0 (StataCorp).

The level of urban development differs with Tier 1, 2, 3, and 4 cities; the disposable income of residents in the designated cities and the medical resources accessible (hospitals and doctors) vary as well (see Table 2). This may lead to differences in the preference for telehealth solutions.

**Table 2:** Disposable income in Shenzhen, Hangzhou, Wuhan, and Yichang (source: CEIC, 2020; National bureau of statistics, 2020).

Category	City	GDP^a^ in 2019 (billion US$)	Disposable and discretionary income (US$)
Tier 1	Shenzhen	422.875	9818.83
Tier 2	Hangzhou	241.425	10357.65
Tier 3	Wuhan	254.744	8120.17
Tier 4	Yichang	70.05	4518.5

^a^GDP: gross domestic product.

Shenzhen was chosen because it is the headquarter of Ping An Technology. Ping An Technology has worked with the government of Shenzhen and other stakeholders to provide electronic medical insurance schemes. Residents in Shenzhen can now use the Ping An Good Doctor app to buy complementary insurance in addition to the basic medical insurance schemes and get refunded online.

Hangzhou was chosen, as it is the city where Alibaba is headquartered. During the interview with Alihealth, the fact that 80% of all primary health care facilities in Zhejiang Province are now equipped with artificial intelligence–assisted image recognition systems was mentioned.

Wuhan was chosen, as it is an important hub in Central China where the population is growing rapidly in recent years. Recently, the Wuhan Municipality has launched several programs promoting the internet + home care initiative for the elderly. There are several exploratory projects running in different districts in Wuhan such as in the Dongxihu and Wuchang districts. There have been several models proposed and tested in Wuhan for elderly care such as the community embedded model, centralization model, and combinations of the proper centralization and decentralization models. Services provided to the elderly focus on assisted food service, assisted cleaning service, assisted nursing and medical service, and long-distance care.

Yichang was chosen, as the level of aging population in Yi Chang is higher than the national average. Aging was measured by the percentage of people over 60 years old in the entire population and the percentage of people over 80 years old in the elderly population. The Yichang municipality is currently developing community-based care centers and rural cooperative elderly care centers. The Yichang municipality established the telehealth solution platform for elderly care in 2019. Using the platform, in 2020, the tele-elderly-care (translated from Chinese) services were expected to reach all townships in Yichang and cover over 50% of all elderly people.

### Theoretical Model and Hypothesis

To evaluate users’ willingness to use telehealth solutions, 3 hypotheses were formulated; in addition, the model considers the effects of demographic factors such as age, education background, income, health status, and living habits such as regular social activity and regular exercise. Table 3 describes the theoretical model built to assess users’ willingness to use telehealth solutions, the hypothesis, and the variables involved in the model. The theoretical model consists of 2 parts; the first part assesses the Likert scale factors and their correlation with the users’ willingness; the second part assesses demographic factors and their impact on the 3 factors and the willingness to use telehealth solutions.

Considering that the dependent variable, namely the willingness to use telehealth solutions, is an ordered discrete variable, the ordered logit model is used for regression. The impact of each factor was assessed by designing 4 models.

Y = βF_1_ + γZ + ε **(1)**

Y = βF>_2_ + γZ+ ε **(2)**

Y = βF_3_ + γZ + ε **(3)**

Y = β_1_F_1_ + β_2_F_2_ + β_3_F_3_ + γZ + ε **(4)**

Model (1) is used to test the impact of Factor 1, and models (2) and (3) are used to test the impact of Factors 2 and 3. Model (4) considers the influence of the above 3 factors.

*Y* represents the designated value for the willingness of participants to use telehealth solutions. In the original questionnaire, the question assigned the preference level as from 1 to 7 (1=preference for traditional health solutions [face-to-face communication], 4=neutral, and 7=preference for telehealth solutions). *Z* represents the control variables such as demographic factors, including the living city, age, gender, education level, health condition, income, living situation, and lifestyle variables (regular exercise and regular social activity) of the participants.

**Table 3:** Hypotheses and corresponding variables in the model.

Factor		Hypothesis		Corresponding question in the questionnaire
**Factor 1**				
	1.1 Social Influence	1.1 Social influence (friend and family opinions) has an impact on the willingness to use telehealth solutions.	Q28, Likert scale value: 1-7	
	1.2 Price	1.2 The price of telehealth solutions has an impact on the willingness to use telehealth solutions.	Q24, Likert scale value: 1-7	
	1.3 Design and brand	1.3 The brand and design of telehealth solutions have an impact on the willingness to use telehealth solutions.	Q25, Likert scale value: 1-7	
	1.4 Privacy risk	1.4 The privacy risk associated with the use of telehealth solutions has an impact on the willingness to use these solutions.	Q23, Likert scale value: 1-7	
	1.5 Private insurance or business insurance coverage	1.5 Private or business insurance plan coverage has an impact on the willingness to use telehealth solutions.	Q18, Likert scale value: 1-7	
**Factor 2: Health-related motivation factors**				
	2.1 Lower health risk	2.1 The belief that telehealth solutions can lower health risk is positively related to the willingness to use telehealth solutions.	Q14, Likert scale value: 1-7	
	2.2 Raise health awareness	2.2 The belief that telehealth solutions can raise health awareness is positively related to the willingness to use telehealth solutions.	Q13, Likert scale value: 1-7	
	2.3 Lack of community health care for patients	2.3 The belief that telehealth solutions can amend the gap in the lack of community health care for patients has an impact on the willingness to use telehealth solutions.	Q22, Likert scale value: 1-7	
	2.4 Unstable doctor-patient relationship	2.4 The belief that telehealth solutions can help improve doctor-patient relationship has an impact on the willingness to use telehealth solutions.	Q15, Likert scale value: 1-7	
**Factor 3: Trust**				
	3. Data accuracy	3. The accuracy of the data collected by telehealth solutions has an impact on the willingness to use telehealth solutions.	Q22, Likert scale value: 1-7	
**Control variables**				
	Residence city	The residence city of the participants has an impact on Factors 1, 2, and 3 and their willingness to use telehealth solutions.	Q3, 1=Shenzhen, 2=Hangzhou, 3=Wuhan, and 4=Yichang	
	Gender	The gender of the participants has an impact on Factors 1, 2, and 3 and their willingness to use telehealth solutions.	Q30, 0=female and 1=male	
	Education	The education level of the participants has an impact on Factors 1, 2, and 3 and their willingness to use telehealth solutions.	Q31, 1=Primary school education (0-6 years), 2=junior/senior high school education (6-12 years), 3=vocational training (12-15 years), 4=college education (15-18 years), and 5=graduate school education (>=18 years)	
	Income	The monthly household income of the participants has an impact on Factors 1, 2, and 3 and their willingness to use telehealth solutions.	Q29, 1=no fixed income, 2=monthly household income <=US $785.23, 3=monthly household income >US $785.23 but <=US $1570.45, 4=monthly income >US $1570.45) but <=US $4711.35, and 5=monthly income >US $4711.35, original value in RMB, 1 USD=6.37 RMB	
	Health status	The self-reported health status of the participants has an impact on Factors 1, 2, and 3 and their willingness to use telehealth solutions.	Q11, 1=self-reported healthy, 2=suboptimal healthy, 3=with chronic disease having no significant impact on life quality, and 4=have chronic disease with significant impact on life quality	
	Preferred living status	The preferred living situation of the participants has an impact on Factors 1, 2, and 3 and their willingness to use telehealth solutions.	Q4, 1=Prefer living alone, 2=prefer living with partner, 3=prefer living with children, and 4=prefer living with children or grandchildren	
	Regular exercise	Participants engaging or not engaging in regular exercise has an impact on Factors 1, 2, and 3 and their willingness to use telehealth solutions.	Q9, 1=Socialize regularly and –1=do not socialize regularly	
	Regular social activity	Participants engaging or not engaging in regular social activities has an impact on Factors 1, 2, and 3 and their willingness to use telehealth solutions.	Q10, 1=Exercise regularly and –1=do not exercise regularly	

## Results

### Health Status of Survey Participants

Based on the self-identified responses from the subjects, the following categories were created to identify their health status: healthy, suboptimal healthy, with chronic disease, and self-identified healthy. Then, more detailed data, such as the type of chronic disease and the number of chronic diseases of the survey participants, were analyzed.

Among the 390 participants, 117 (30%) reported having 1 chronic disease (30%), 64 (16.4%) reported having 2 chronic diseases (16.4%), and 47 (12.05%) responded as having 3 chronic diseases (12.05%). Further, 17 participants (4.36%) stated having 4 chronic diseases (4.36%), 7 (1.79%) reported having 5 chronic diseases, 6 (1.53%) reported having 6 chronic diseases, and 2 (0.5%) reported having communicable and chronic diseases (0.5%). Furthermore, 110 participants (28%) reported having no chronic diseases.

### Descriptive Statistics

In this section, the qualitative analytical results are presented. All the survey participants are over 50 years old because the survey intends to collect information on elderly users’ needs in the next 5 to 10 years, as shown in Table 4. Among the 390 participants providing valid answers, 160 (41.03%) indicate that they are more willing to use traditional health care solutions; 167 (42.82%) indicate that they are willing to use telehealth solutions, whereas 51 (13.07%) show neutral willingness.

Among the 390 participants, 112 (28.7%) are aged 51 to 60 years; another 112 participants (28.7%) are aged 61 to 70 years. Further, 110 participants (28.2%) are aged 71 to 80 years is. There are 43 participants (11.4%) over 80 years old; the number of participants in this group is less than that in the other 3 age groups, as data subject recruitment was limited by the physical conditions of the individuals in this age group.

Moreover, 67.7% (264/390) of the participants often use telehealth solutions to monitor health status. Most survey participants (246/390, 63.1%) received 6 to 12 years of education, followed by 83 participants (21.3%) who went to elementary school. Given the survey candidate recruitment conditions for the elderly aged above 50 years, the education level of the participants is in line with the reality.

**Figure 1:**
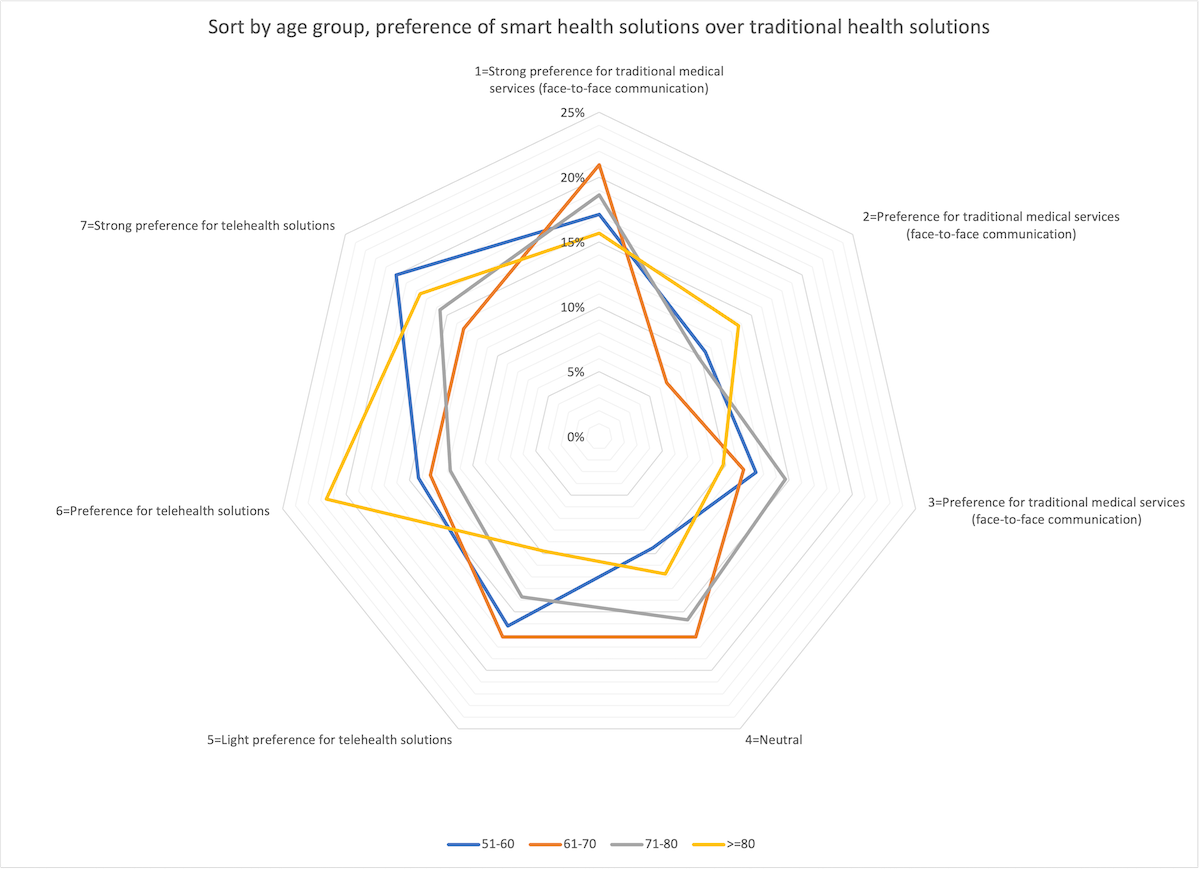
Willingness to use telehealth solutions sorted by age group.

The distribution diagram in Figure 1 shows that participants in the age group of 51 to 60 years and those aged above 80 years show a strong willingness or a willingness to use telehealth solutions. This may be because people in the age group of 50 to 60 years are more familiar with technology, whereas those above 80 years cannot physically attend in-person doctor visits.

**Table 4:** Demographic characteristics of participants (N=390).

Characteristic			n (%)
**Gender**			
	Male	224 (57.4)	
	Female	166 (42.6)	
**Age (years)**			
	51-60	112 (28.7)	
	61-70	112 (28.7)	
	71-80	110 (28.2)	
	>=80	56 (14.4)	
**Residence city**			
	Shenzhen	97 (24.9)	
	Hangzhou	95 (24.4)	
	Wuhan	108 (27.7)	
	Yichang	90 (23.1)	
**Household income (US$, original value in RMB, $1 = 6.37 RMB)**			
	No fixed monthly income	21 (5.4)	
	≤785.23	84 (21.5)	
	785.23-1570.45	186 (47.7)	
	1570.45-4711.35	88 (22.6)	
	≥4711.35	11 (2.8)	
**Frequency of using telehealth solutions**			
	Often	264 (67.7)	
	Occasionally	82 (21)	
	Rarely	44 (11.3)	
**Education level**			
	Primary school (1-6 years)	83 (21.3)	
	Junior or high school (6-12 years)	246 (63.1)	
	Vocational training (12-15 years)	31 (7.9)	
	College graduate (15-18 years)	29 (7.4)	
	Graduate School (>=18 years)	1 (0.3)	
**Health status**			
	Healthy	145 (37.2)	
	Suboptimal healthy	99 (25.4)	
	With minor chronic disease	132 (33.8)	
	With major chronic disease affecting life quality	14 (3.6)	
**Living situation**			
	Living alone	47 (12.1)	
	Living with partner	156 (40)	
	Living with children	177 (45.4)	
	Living with grandchildren	4 (1)	
**Health insurance status**			
	None	8 (2.1)	
	Basic resident or employee medical insurance	309 (79.2)	
	Private insurance	7 (1.8)	
	Other social insurance schemes	21 (5.4)	
	Public servant insurance	38 (9.7)	
	Unknown	7 (1.8)	

**Figure 2:**
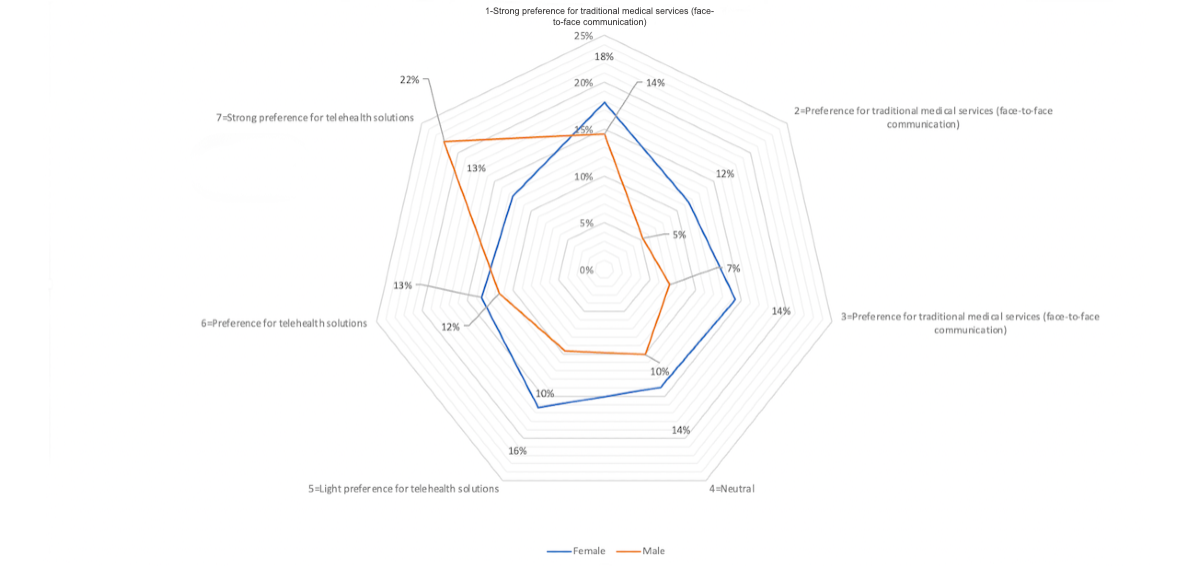
Willingness to use telehealth solutions based on gender.

Among the 390 users surveyed, there are 224 males and 166 females, accounting for 57.4% and 42.6% of the total number of participants, respectively; the proportion of male users is higher than that of female users (as observed in Table 4). Figure 2 suggests that female users are willing to use traditional medical solutions, whereas male users are strongly willing to use telehealth solutions.

**Figure 3:**
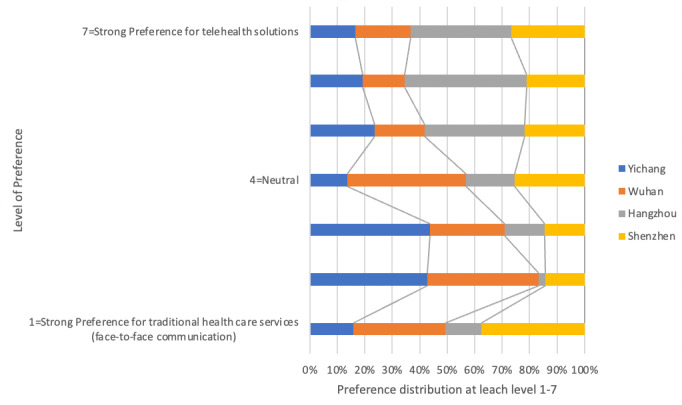
Willingness to use telehealth solutions in Yichang, Wuhan, Hangzhou, and Shenzhen.

In accordance with the study design, survey participants are evenly distributed in the 4 cities. The number of data samples obtained in Shenzhen, Hangzhou, Wuhan, and Yichang are 97, 95, 108, and 90, accounting for 24.9%, 24.4%, 27.7%, and 23.1%, respectively, of the 390 data subjects. Figure 3 suggests that in Shenzhen and Wuhan, the percentage of participants showing preference for telehealth solutions is higher than that in Hangzhou and Yichang.

**Figure 4:**
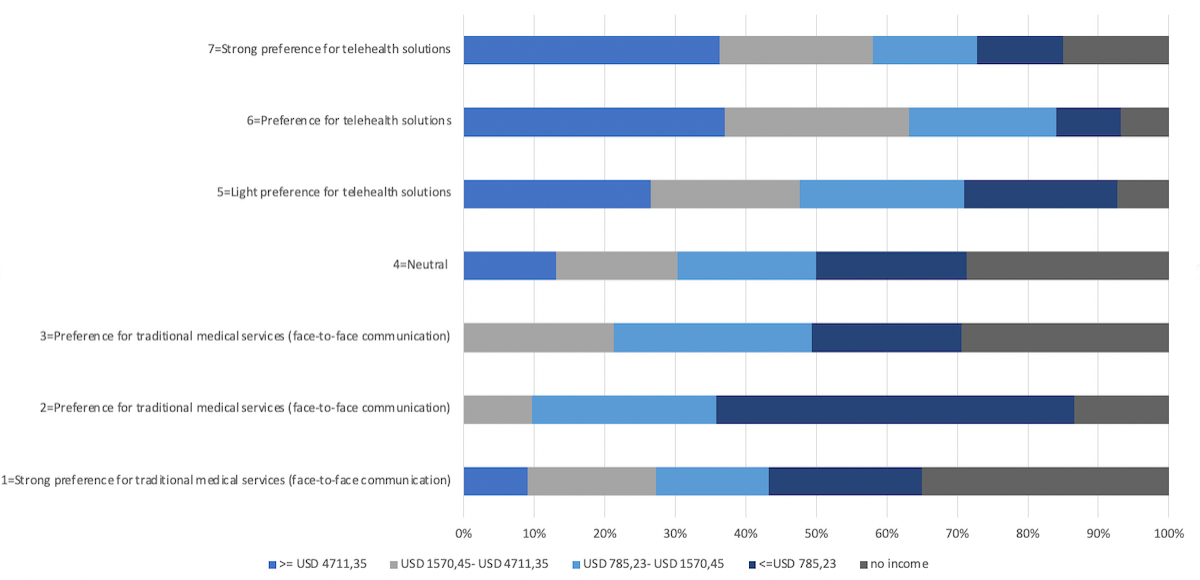
Willingness of the participants in the 5 income groups to use telehealth solutions.

The distribution of income follows the bell curve, with approximately half (186/390, 47.7%) of the sample’s monthly household income falling between US$ 785.23 and US$ 1570.45; the proportions of the sample with household monthly incomes less than or equal to US$ 785.23 and more than or equal to US$ 4711.35 account for only 5.4% (21/390) and 2.8% (11/390), respectively. The willingness to use telehealth solutions increases with the monthly income as well. Figure 4 points out that in the >=US$ 4711.35 income group, the preference is mainly neutral and above neutral. The lower the income, the higher the percentage of the surveyed data subjects showing strong preference for traditional health solutions. This can be observed among the no income and <=US$ 785.23 income groups.

In terms of using telehealth solutions for monitoring sleep and nutrition intake, the percentage of users who are currently using telehealth solutions for sleep monitoring and nutrition monitoring are respectively 23.07% (90/390) and 26.15% (102/390).

**Figure 5:**
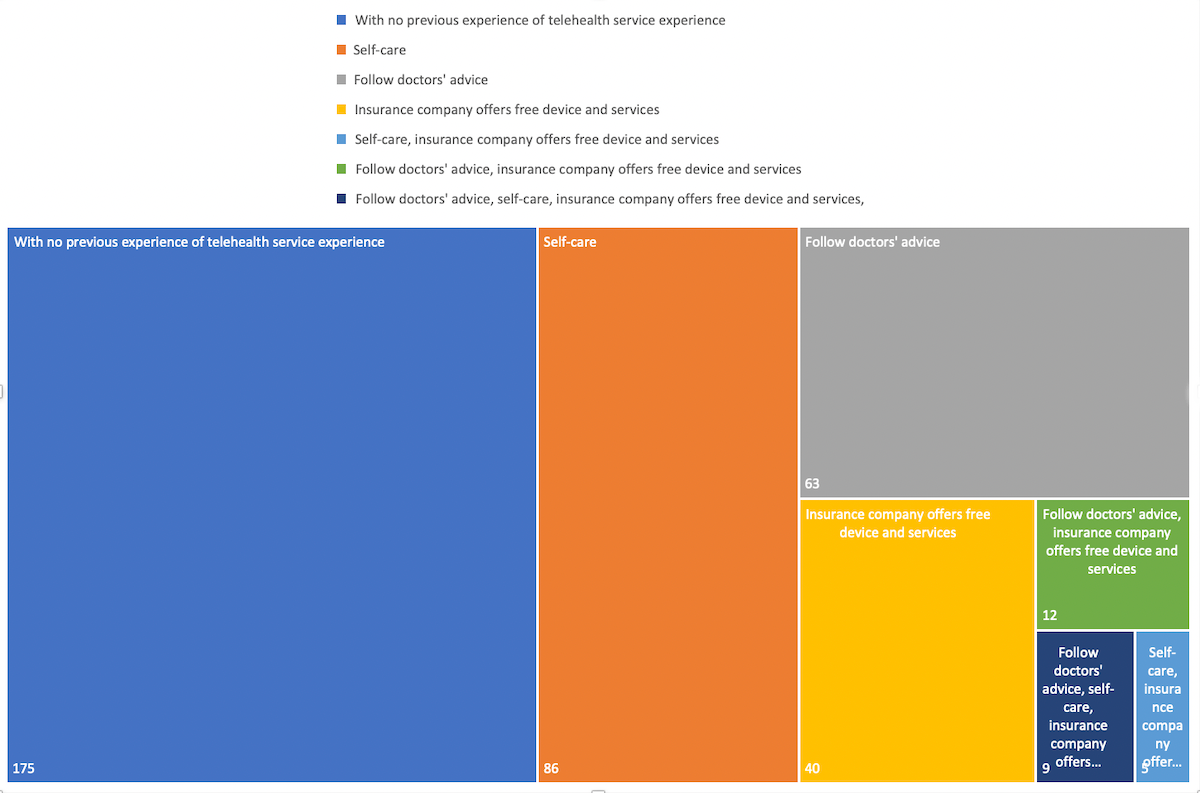
Reasons for using telehealth solutions.

Figure 5 indicates that the major reasons for using telehealth devices are self-care, following the doctor’s advice, and the free devices and services offered by insurance companies in China or a mix of these 3 reasons.

The factors affecting the willingness to use telehealth solutions are ranked by the mean of each variable (Likert scale: 1-7, 1=no impact, 4=neutral, and 7=with an impact), as shown in Table 5. Among the 10 factors, 6 have means more than 4, suggesting that these factors influence the preference for telehealth solutions. The top 4 motivations are lowering health risks, raising health care awareness, lack of community medical services, and following the doctor’s advice; these variables comprise Factor 1.

Factor 2 consists of the price, privacy risk, social influence, design and brand of the solution, and the participants’ coverage with insurance plans. The mean value of these variables is close to neutral or less than 4, suggesting that survey participants in general do not believe that these factors influence their willingness to use telehealth solutions.

The accuracy of the data (Factor 3) collected through telehealth solutions is also a key factor. Compared with traditional medical instruments and equipment having the shortcoming of inaccurate data reading, telehealth solutions collect more accurate health data. However, most doctors and hospitals still do not trust data collected from telehealth solutions and do not use these data sources as the basis for diagnosis or treatment. This makes it difficult for users to trust the devices used for collecting health data and monitoring health status.

**Table 5:** Ranking of factors affecting the willingness to use telehealth solutions among elderly users.

Factors	Ranking	Mean	SE
Lowering health risk	1	5.96	1.672
Raising health awareness	2	5.85	1.676
Lack of community health care service	3	5.77	1.721
Following doctors’ prescriptions	4	5.27	2.032
Price of the solution	5	4.37	2.462
Data accuracy	6	4.07	2.314
Design of the solution	7	3.72	2.498
Privacy risk	8	3.70	2.342
Social influence	9	3.44	2.540
Free device offered by insurance companies	10	2.77	2.157

### Modeling Process

This section presents the quantitative analytical results.

To avoid heterogeneity issues, the Kaiser–Meyer–Olkin (KMO) and Bartlett test was conducted to examine the correlation between the Likert scale variables; the KMO score of 0.796 suggests that the sample is adequate for factor analysis. Then, principal component analysis (PCA) was performed to reduce the dimensions of the model and the correlation between variables. With the factor loading for each factor confirmed, the Likert scale variables were then ranked based on the mean value of each variable. The next step was to test if demographic factors influenced the 3 factors identified by PCA. This was confirmed with analysis of variance (ANOVA) and *t* tests.

The modeling process started with a correlation matrix (Pearson correlation and Spearman rank correlation) to test if the data have multicollinearity (see Multimedia Appendix 2). Then, the O-logit model was run using Stata 16.0 along with the control variables. During the final modeling step, 10 participants were randomly selected to determine if the prediction preference scores matched the choices made by the participants.

The KMO and Bartlett test was conducted on 10 Likert scale factors related to survey participant preferences. The KMO coefficient is 0.796 (>0.5) with the Sig. value of 0.000 in the Bartlett sphere being less than 0.05, indicating that there is a certain degree of correlation among the 10 factors. Dimension reduction among the 10 factors was deemed necessary for further analysis.

Factor analysis is a commonly used dimensionality reduction method. PCA and the varimax right-angle rotation method were used to extract 3 principal factors. These 3 principal factors could explain 64.149% of the total variance, with the first, second, and third factors explaining 28.364%, 25.196%, and 10.589% of the total variance, respectively (see Tables 6 and 7). The variables selected had a factor loading greater than 0.5 (See Table 6).

Factor 1 consists of the price (0.812), design (0.738), impact of private insurance coverage (0.713), social influence (0.706), and privacy risk (0.612).

Factor 2, involving health-related motivations, consists of lowering health risk (0.864), raising health awareness (0.818), lack of community health care services (0.771), and following the doctor’s advice or prescription (0.701).

Factor 3, related to the trust for telehealth solutions, consists of the data accuracy variable. Users’ trust levels for telehealth solutions are influenced by whether data collected from wearables or medical devices at home are accepted by doctors and hospitals. Therefore, trust is influenced directly by the data accuracy of the solution (0.762).

ANOVA and *t* tests were conducted for assessing whether the relative importance of the 3 main factors from PCA analysis differ, depending on the city of residence, age, gender, education level, health status, income, living situation, regular social activity, and regular exercise. The results are shown in Table 8.

**Table 6:** Principal component analysis.

Component		Factor loading	Eigenvalue	Variance contribution rate	Cumulative contribution rate
**Factor 1**			2.836	28.364	28.364
	Price	0.812			
	Brand and design	0.738			
	Private insurance coverage	0.713			
	Social influence	0.706			
	Privacy risk	0.612			
**Factor 2: Health-related motivations**			2.520	25.196	53.560
	Lower health risk	0.864			
	Raise health awareness	0.818			
	Lack of community health care service	0.771			
	Unstable doctor-patient relationship	0.701			
**Factor 3: Trust**			1.059	10.589	64.149
	Data accuracy	0.762			

**Table 7:** Total variance explaineda.

Factor	Rotation sums of squared loadings		
	Total	% variance	Cumulative %
1	2.836	28.364	28.364
2	2.520	25.196	53.560
3	1.059	10.589	64.149

^a^Extraction method: PCA.

First, we assumed that the relative importance of factors varies with the residence city. The results of variance analysis support this hypothesis.

The first factor is related to the price, brand, and design associated with the telehealth solutions; factors such as social influence and private insurance coverage are also included.

Second, we assumed that age plays a significant role in determining user preference. In this study, survey participants were divided into 4 age groups, 51-60, 61-70, 71-80, and over 80 years. Considering that 71.79% (280/390) of all the survey participants have chronic diseases, users from this age group may consider the relevant health benefits such as raising health awareness, lowering health risk, and improving access to health care more than other age groups. ANOVA results suggest that the second factor varies with age (*P*=.088). The second factor mainly reflects the belief that telehealth solutions can raise health awareness, lower health risk, improve doctor-patient relationships and amend the gap related to the lack of community health care services.

Gender is also one of the key factors affecting user preference. The hypothesis is that male and female users have a perceived value, perceived risk, and perceived benefit associated with telehealth solutions. Considering the binary factor of gender, the *t* test could verify our hypothesis. The results show that the trust factor is significant at the level of 1%. This suggests that male and female survey participants differ in their trust on the data accuracy risk related to telehealth solutions.

The survey classifies users’ education levels by years into five categories: primary school (1-6 years), high school (6-12 years), vocational school (12-15 years), college education (15-18 years), and postgraduation (>=18 years). Our hypothesis is that Factors 1, 2, and 3 differ across different education levels. However, the ANOVA results reject our hypothesis. With data suggesting that 84.4% of all survey participants have high school or primary school education, the conclusion is that survey participants with less than 15 years of education show no difference in Factors 1, 2, and 3.

The health status of survey participants is divided into four categories: self-reported healthy, suboptimal health status, with chronic disease (does not affect life quality), and with chronic disease (affects life quality). The variance analysis results support our hypothesis. The third factor, trust over data accuracy regarding telehealth solutions, is statistically significant and is affected by the health status of survey participants.

ANOVA results suggest that household income has a statistically significant effect on Factor 2, consisting of factors pertaining to health-related motivations. Families with high household incomes can bear the cost of using telehealth solutions, thereby benefiting from active self-health management. Users in the lower household income group pay more attention to factors such as the price of telehealth solutions, often ignoring the need for active health management. Factor 2 varies among different income groups.

Trust over data accuracy regarding telehealth solutions (Factor 3) is also affected by whether survey participants live with their children or grandchildren. The survey participants’ living situations are categorized as prefer living alone, prefer living with spouse, prefer living with children, and prefer living with grandchildren. Usually, it is the children and grandchildren living with their parents or grandparents who pay for telehealth solutions and teach their parents and grandparents to use such solutions. The elderly people thus benefit from living with their children or grandchildren and trust the telehealth solutions more than those who live alone or with spouses only.

**Table 8:** One-way analysis of variance and two-sample *t* test.

Hypothesis testing and variance analysis value			Factor 1		Factor 2	Factor 3
**Residence city**						
	*F* value (*df1, df2*)	5.718 (3, 386)		2.245 (3, 386)		4.075 (3, 386)
	*P* value	.001		.083		.007
**Age**						
	*F* value *(df1, df2)*	0.467 (3, 386)		2.195 (3, 386)		0.172 (3, 386)
	*P* value	.71		.088		.92
**Gender**						
	*F* value *(df1, df2)*	0.074 (1, 388)		2.128 (1, 388)		7.570 (1, 388)
	*P* value	.79		.15		.006
**Education**						
	*F* value *(df1, df2)*	1.186 (4, 385)		0.180 (4, 385)		1.374 (4, 385)
	*P* value	.32		.95		.24
**Health condition**						
	*F* value *(df1, df2)*	1.494 (3, 386)		1.128 (3, 386)		3.468 (3, 386)
	*P* value	.22		.34		.02
**Income**						
	*F* value *(df1, df2)*	1.261 (4, 385)		4.109 (4, 385)		1.436 (4, 385)
	*P* value	.29		.003		.22
**Living situation**						
	*F* value *(df1, df2)*	1.216 (5, 384)		1.136 (5, 384)		2.665 (5, 384)
	*P* value	.30		.34		.022
**Regular social activity**						
	*F* value *(df1, df2)*	5.998 (1, 388)		2.508 (1, 388)		2.083 (1, 388)
	*P* value	.015		.11		.15
**Regular exercise**						
	*F* value *(df1, df2)*	4.726 (1, 388)		3.963 (1, 388)		0.605 (1, 388)
	*P* value	.03		.047		.44

The *t* test results suggest that regular social activity has a statistically significant effect on Factor 1. Peer pressure from regular social interaction may encourage users to choose telehealth solutions over social influence, brand and design, and insurance plans. Survey participants with poor physical conditions often lack social activity and are subject to less social influence when it comes to using telehealth solutions.

Factors 1 and 2 also differ in terms of whether users exercise regularly. Survey participants exercising regularly are more health conscious and more willing to spend on telehealth solutions such as wearables and believe that telehealth solutions may raise health awareness, lower health risks, amend the gap in community health care and ensure health continuity by improving unstable doctor-patient relationships.

#### Factor 1

Ordered logit regression results suggest that Factor 1 has no statistically significant impact on the preference for telehealth solutions (See Table 9, rows 1 and 3, columns 1 and 4). Therefore, hypothesis 1 is rejected.

**Table 9:** Ordered logit regression.

			(1)		(2)		(3)		(4)
			y		y		y		y
**Factor 1**									
	Coefficient	–0.0227 (*P*=.81)		—^a^		—		–0.0687 (*P*=.47)	
	*t* value (*df)*	–0.2449 (379)		—		—		–0.7299 (377)	
**Factor 2**									
	Coefficient	—		0.4628 (*P*<.001)		—		0.4821 (*P*<.001)	
	*t* value (*df*)	—		4.7735 (379)		—		4.9667 (377)	
**Factor 3**									
	Coefficient	—		—		–0.2554 (*P*=.007)		–0.2856 (*P*=.003)	
	*t* value (*df*)	—		—		–2.7108 (379)		–3.0028 (377)	
**Living city**									
	Coefficient	–0.0235 (*P*=.80)		–0.0463 (*P*=.61)		–0.0062 (*P*=.95)		–0.0201 (*P*=.83)	
	*t* value (*df*)	–0.2604 (379)		–0.5109 (379)		–0.0688 (379)		–0.2208 (377)	
**Age**									
	Coefficient	0.0151 (*P*=.88)		–0.0126 (*P*=.90)		–0.0018 (*P*<.001)		–0.0354 (*P*=.72)	
	*t* value (*df*)	0.1553 (379)		–0.1291 (379)		–0.0180 (379)		–0.3599 (377)	
**Gender**									
	Coefficient	0.2882 (*P*=.13)		0.3820 (*P*=.046)		0.2349 (*P*=.22)		0.3426 (*P*=.075)	
	*t* value (*df*)	1.5150 (379)		1.9995 (379)		1.2331 (379)		1.7801 (377)	
**Education**									
	Coefficient	0.1317 (*P*=.28)		0.1232 (*P*=.31)		0.1565 (*P*=.20)		0.1432 (*P*=.25)	
	*t* value (*df*)	1.0778 (379)		1.0082 (379)		1.2692 (379)		1.1548 (377)	
**Health status**									
	Coefficient	0.0743 (*P*=.48)		0.1365 (*P*=.20)		0.1105 (*P*=.30)		0.1881 (*P*=.084)	
	*t* value (*df*)	0.7034 (379)		1.2821 (379)		1.0352 (379)		1.7290 (377)	
**Income**									
	Coefficient	0.3707 (*P*=.001)		0.2919 (*P*=.01)		0.3908 (*P*=.001)		0.3124 (*P*=.006)	
	*t* value (*df*)	3.3153 (379)		2.5934 (379)		3.4734 (379)		2.7589 (377)	
**Living situation**									
	Coefficient	0.0545 (*P*=.66)		0.0392 (*P*=.75)		0.0446 (*P*=.72)		0.0176 (*P*=.89)	
	*t* value (*df*)	0.4400 (379)		0.3158 (379)		0.3595 (379)		0.1403 (377)	
**Regular socialization**									
	Coefficient	0.1871 (*P*=.069)		0.1714 (*P*=.097)		0.1745 (*P*=.09)		0.1477 (*P*=.15)	
	*t* value (*df*)	1.8191 (379)		1.6619 (379)		1.6979 (379)		1.4264 (377)	
**Regular exercise**									
	Coefficient	–0.0572 (*P*=.64)		–0.0865 (*P*=.48)		–0.0739 (*P*=.55)		–0.1112 (*P*=.37)	
	*t* value (*df*)	–0.4688 (379)		–0.7035 (379)		–0.6018 (379)		–0.8957 (377)	
N			390		390		390		390
Pseudo *R*^2^			0.0169		0.0322		0.0217		0.0384

^a^Not applicable.

#### Factor 2

Hypothesis 2 suggests the correlation between the willingness to use telehealth solutions and the health-related reasons. The regression coefficients of Factor 2 in Models 2 and 4 in Table 9 are positive at the significance level (*P*=.01). Hence, hypothesis 2 is valid.

#### Factor 3 and Preference

Hypothesis 3 assumes that data accuracy risk has a significant impact on the preference of elderly users. Table 9 suggests that the belief in the accuracy of the data collected by telehealth solutions is negatively related to the preference for telehealth solutions in Models 3 and 4. The cut variable suggests there are 7 categories of the dependant variable, where cut 1 puts the category at the lower end when y equals to 0.

To validate the model, 10 samples were randomly selected from the 390 participants to predict the probability of each participant's preference for telehealth solutions. The prediction made by the model is compared with the answer in the questionnaire for validation. Taking the first randomly selected sample as an example, the model suggests that the survey participant is most likely to choose 1 (1=preference for traditional health solutions, 4=neutral, and 7=preference for telehealth solutions). The model suggests that the users' preference for telehealth solutions is low; this is consistent with the users’ actual choice (Y=1). Among the 10 selected samples, the preferences of 8 were successfully predicted, as observed in Table 10. With a prediction rate of 80%, the model is validated. The possibilities of the respondents choosing different categories of preferences (1-7) are denoted as p1 to p7, and the highest possibility (in italics) is the respondent’s choice.

**Table 10:** Model validation^a^.

No.	p1	p2	p3	p4	p5	p6	p7	Y	Prediction results
1	*0.336*	0.153	0.149	0.127	0.107	0.070	0.058	1	yes
2	*0.437*	0.158	0.136	0.104	0.079	0.049	0.039	1	yes
3	*0.174*	0.111	0.139	0.153	0.163	0.131	0.129	1	yes
4	0.127	0.088	0.121	0.148	*0.178*	0.161	0.177	7	no
5	0.135	0.093	0.124	0.150	*0.176*	0.155	0.167	7	no
6	0.052	0.042	0.067	0.101	0.164	0.212	*0.362*	7	yes
7	0.044	0.036	0.059	0.091	0.154	0.213	*0.402*	7	yes
8	0.130	0.090	0.122	0.148	*0.177*	0.159	0.173	5	yes
9	*0.202*	0.122	0.145	0.152	0.153	0.117	0.110	1	yes
10	0.076	0.058	0.088	0.124	0.179	0.199	*0.276*	7	yes

^a^p1 to p7: possibilities of the respondents choosing different categories of preferences.

## Discussion

### Principal Findings

Digitalization of the health care system has been rapidly accelerated by COVID-19. Because of social distancing and the highly communicable nature of the disease, the use of telehealth solutions grew exponentially, with expenditure on such solutions increasing as well. The high number of COVID-19 patients has consumed hospital and medical resources rapidly, depriving medical care for many patients with chronic diseases. The importance of using telemonitoring and telehealth solutions inside and outside the hospital setting has become more important than ever.

This study analyzed questionnaire data collected on factors related to the preference for telehealth solutions in Shenzhen, Hangzhou, Wuhan, and Yichang. The preference is related to the following factors: F1-perceived value of telehealth solutions related to product price, design, and social influence; F3-perceived risk for telehealth solutions related to trust over telehealth solutions; and F2-perceived benefits for telehealth solutions in self-care and health management. ANOVA and *t* tests were conducted to verify the influence of demographic variables on Factors 1, 2, and 3. The ordered logit regression results suggest that the perceived value (F1) has no significant impact on the preference for telehealth solutions, indicating the homogeneity of current consumer-facing telehealth solutions. Perceived benefits in self-care and health management (F2) are positively related with the preference for telehealth solutions, and F3 (trust over data accuracy) is negatively correlated with the preference for telehealth solutions.

The preference distribution graph (Figure 2) suggests that females are more conservative than males in their preference for telehealth solutions. This may be related to their income, health education, and tendency to socialize. This will make female users more willing to communicate with doctors face to face and use traditional health solutions.

Reasons behind the differences in the preference for telehealth solutions among residents from different cities can be explained based on infrastructure differences, such as the smart health initiative in Shenzhen and Wuhan and the concentration of hospitals and other medical resources in Wuhan. Shenzhen and Wuhan started early in their big data + health initiative, whereas other cities started later. The big data + health initiative provides the necessary digital infrastructure (hospital information system) for health system digitalization including the digitalization of hospitals and the connection of primary health service clinics.

The preference for telehealth solutions varies by income, and this may exist because communications with doctors are part of social activity; such social activities are strongly related to health education, social influence, and health insurance coverage. The lower income groups have less insurance coverage and less health education; they have a strong preference for seeing doctors in person and spending hours at hospitals because they have fewer other social activities. Higher income groups have better health education, wider health insurance coverage, and considerably less willingness to spend time at hospitals. Therefore, as they can afford telehealth solutions, based on the premise that telehealth solutions are not covered by the social (employee or resident) medical insurance schemes in China, the higher income groups are more willing to use telehealth solutions rather than wait at hospitals.

It is still necessary to address the data interoperability issue between hospitals and the primary health service clinics to ensure patients’ care continuity; this may also help reduce the concentration of patients at hospitals and divert them back to primary care institutions. After all, doctors at level-3 hospitals have no time for helping outpatients to address their lifestyle problems once they leave the hospital. This gap leaves room for community care centers to step in and advise patients and monitor them regularly. The vacuum for community care centers can be filled in by telehealth solutions for patient self-care.

There are more telehealth solution providers in Shenzhen and Hangzhou, as well as high-quality hospitals. Doctors and nurses are more acceptable for telehealth solutions in large cities such as Shenzhen and Hangzhou. A wide variety of well-designed and affordable telehealth solutions are available in Shenzhen and Hangzhou. With the fast-paced lifestyle in these 2 cities, elderly (aged 50 years and above) users are more willing to use telehealth solutions. Considering the differences in disposable monthly income, it is more likely that residents in Shenzhen and Hangzhou enjoy the coverage of private insurance. Users with private health insurance coverage are more likely to believe that the coverage of telehealth solutions has an impact on the willingness to use telehealth solutions (whether positive or negative).

The homogeneity of DTC telehealth solutions can lead to indifference in users over factors such as the price, design, privacy risk, and brand and design of telehealth solutions. Currently, telehealth solution providers focus on providing heterogeneous solutions at lower prices. This lowers product profits and deters progress made in data interoperability and the acceptance of telehealth solutions by health care service providers. Some solution providers choose to cut core component configurations to reduce costs, thus failing to guarantee the quality of the solution. Some solution providers have actively marketed their products by offering installments and interest-free loans to attract users. Although promotion and marketing remain important, equipment manufacturers may consider improving the competitiveness of their products by promoting the medical value of their solutions, integrating the solution with the electronic health record system, providing noninvasive monitoring equipment, improving data accuracy, and providing privacy protection.

The findings of the study suggest that users do believe that telehealth solutions can improve health awareness, reduce health risk, mend the gap in community health care services, and improve care continuity, thus having a positive impact on the willingness to use telehealth solutions. Meanwhile, doctors’ suggestions and prescriptions play a role in driving users to choose telehealth solutions over traditional health solutions as well. Elderly (aged 50 years and above) users have a strong demand for self-care and health management anytime and anywhere. Telehealth solutions that are easy to operate, carry, and understand can effectively meet the demand of elderly users for self-care and health management. Moreover, with the global need for qualified clinicians [20], the demand for telehealth solutions is expected to increase.

Compared with traditional health management methods, telehealth solutions offer convenient ways for maintaining health records and health care management at home. However, elderly users do not trust data collected through telehealth solutions. For example, elderly patients with hypertension prefer to go to the doctor to check their blood pressure instead of using the Bluetooth-connected blood pressure monitor at home. With the lack of integration of EHR systems, doctors cannot use the discontinuous data, even if the data collected by telehealth devices are relatively more accurate. There are also technical trust challenges regarding whether the algorithms are trained using accurate data representative of the potential user group. Human trust in the usability of the system, and the regulatory trust issues related to the ethical, legal, and social implications of the use of AI in health care are also important aspects to address [21]. With elderly users, it is extremely important to address the system usability issue of telehealth solutions and build human-level trust.

In the stakeholder interview stage, an interviewed doctor stated that elderly people with chronic diseases are more willing to go to the doctor for blood pressure measurement. The willingness to use telehealth solutions is affected by the users' health condition. For instance, telehealth solutions can provide users with a large amount of real-time personal health data, such as the heart rate, blood pressure, blood sugar, and other health indexes. For users with chronic diseases, although the data collected by telehealth solutions have certain reference values, doctors either have no access to the data or do not trust the data collected at home. With little or no integration with the health care system, elderly users do not trust the data collected through telehealth solutions.

### Conclusions

Many existing telehealth solutions have similar features, prices, and designs. However, doctors often fail to use the data collected from telehealth solutions in making diagnosis and treatment decisions. They often use data collected from telehealth solutions as a reference but rarely base their decision on the daily data trends. Faced with increasing amounts of data from patients, resolving the data interoperability challenges between telehealth solution systems and hospital EHR systems seems more urgent than ever. Further research can focus on data interoperability between the EHR systems and telehealth solutions. The medical value of telehealth solutions can improve if doctors could interpret data collected from telehealth solutions; furthermore, if doctors could diagnose, provide treatments, and adjust health care management plans based on such data, telehealth solutions can be included in insurance packages, making them more accessible.

There are hurdles to building trust for using telehealth solutions and AI in health care. Future research can also be extended to address such challenges by analyzing how to improve the transparency of algorithms by disclosing the data source and how the algorithms were built.

Owing to the limited scale of the questionnaire study (N=390), this paper only serves as a reference for exploring the implementation of telehealth solutions among elderly users in the next 5 to 10 years. The study was carried out in 4 Chinese cities (Shenzhen, Hangzhou, Wuhan, and Yichang), where the public health system differs in many ways from the health care systems in the United States or Europe. Therefore, the study is limited in scale with a sample size of 390 and remains an exploratory stage study. Further work can be done on the preference for telehealth solutions post-COVID and the changes in business models for telehealth solutions.

## Appendix

Multimedia Appendix 1 English version of the questionnaire.

Multimedia Appendix 2 Pearson test and Spearman rank correlation test.

## Abbreviations

ANOVA: analysis of variance
DCE: discrete choice experiment
KMO: Kaiser–Meyer–Olkin
PCA: principal component analysis
