# Supplementary material for: Assessing Elderly User Preference for Telehealth Solutions in China: Exploratory Quantitative Study
Source: JMIR Mhealth Uhealth. 2022 Jan 12;10(1):e27272. doi: 10.2196/27272 (PMC8792775; doi:10.2196/27272)
Supplement: Multimedia Appendix 1 [file mhealth_v10i1e27272_app1.pdf]

HEalth related Activity Recognition system based on IoT
An interdisciplinary training program for young researchers
www.heart-itn.eu

HEART PROJECT

This project has received funding from the European Union's Horizon 2020 research and innovation programme under the Marie Skłodowska-Curie grant agreement No 766139

Smart health solutions - factors affecting users' intention to use questionnaire research

Hi, I am Nuoya Chen, an early stage researcher for the Marie Curie Heart Project. The HEART project is about health related activity recognition system based on Internet of things. My research topic is on strategic marketing, predicting market trends and gaps where the Internet of Healthcare Things can fill in the next 5 to 10 years. Currently, I am doing some research on user experience and applicable business model for the Internet of Healthcare things. If you are interested in my research, can you please help me to fill out this questionnaire? It will take you 5-10 minutes to go through the questions.

Thank you for your help.

Nuoya Chen

For feedback, please email: c.nuoya@studenti.unimc.it

Sponsor: This project has received funding from the European Union’s Horizon 2020 research & innovation programme under the Marie Skłodowska-Curie – ITN Industrial Doctorate, Grant agreement No. 766139

This questionnaire reflects only the author’s view and the REA is not responsible for any use that may be made of the information it contains.

This project has received funding from the European Union's Horizon 2020 research and innovation programme under the Marie Skłodowska-Curie grant agreement No [illegible]

1. Do you agree to answer this questionnaire and the data collected will be used for scientific research purpose?
   Yes -------------- 1
   No -------------- -1

2. Which one of the following options your age group?
   <= 18 (including 18) ----------------- 1
   19-29 (including 29) ----------------- 2
   30-49 (including 49) ----------------- 3
   >=50 (including 50) ------------------ 4

3. In which city are you a regular resident?

   Shenzhen ---------1

   Hangzhou ---------- 2

   Wuhan ----------- 3

   Yichang ---------- 4

4. Which one of the following options do you prefer?
   Living alone -------- 1
   Living with my partner --------- 2
   Living with my children ------------- 3
   Living with my children and grandchildren -------- 4
   Other _please write down your answer if you choose other

Part I: Basic information regarding the use of smart health management tools

Smart health management tools include smart phone apps (such as Alihealth, Ping An Good Doctor, Chun Yu Doctor, Wedoctor, Yue dong quan, etc), wearables (such as Xiaomi Band, Huawei watch and Apple Watch, etc), home use health management tools (such as PICOOC smart scale, Mi Home i-Health blood pressure monitor, Mi Home Hi-Pee Smart Pee Monitor, Smart Sleep Monitor, Smart devices to improve sleep quality, etc.).Can you please choose the right answer based on your use experiences?

5. Do you use smart health devices(smart phone apps, wearables, home based smart health devices) to monitor sleep (sleep time, deep sleep time) or to improve sleep quality?

   Yes ------------ 1

   No -------------- -1

This project has received funding from the European Union's Horizon 2020 research and innovation programme under the Marie Skłodowska-Curie grant agreement No 766139

6. Do you use smart health management tools (smart phone apps, wearables, home-based smart medical devices, etc) to control nutrition and diet (such as to follow low sugar and low salt diet)?

Yes ------- 1

No ----------- -1

7. How often do you use your smart health management tools (apps, wearables, online medication websites, e-health records, etc)?

Often ----------- 1

Rarely ------------ 0

Never ------------- -1

8. Why do you feel there is a need to use smart health solutions?

To control my health situation thoroughly -------- 1

To follow doctor's prescription ------------ 2

My insurance company give me a free product (device, services) ----------------3

Other reasons (Please indicate your answer if you choose other reasons)

Part II

Please answer the questions based on your understanding of your health and choose the best answer you feel.

9. Do you have regular social activities?

Yes ----------- 1

No ------------ -1

This project has received funding from the European Union's Horizon 2020 research and innovation programme under the Marie Skłodowska-Curie grant agreement No [illegible]

10. Do you exercise regularly?

Yes ----------- 1

No ------------- -1

11. How is your health situation in general?

Healthy -------- 1

Suboptimal health ----------------- 2

With chronic disease (Does not affect daily life) ------------- 3

With chronic disease (Affect daily life) ----------------- 4

12. Do you have one of the following categories of diseases?

Cardiovascular diseases -------------- 1

Diabetes I/II -------------- 2

Hypertension ----------------- 3

Chronical Bowel Disease ----------- 4

Chronical Communicable Disease (Hepatitis B infection) ------------ 5

Rheumatic Arthritis ----------------- 6

Osteoporosis ------------- 7

Eye Diseases ------------- 8

Cancer -------------------- 9

No, I am heathy. / Other _______________ (if you choose other, please write down your answer)

13. Do you believe that smart health solutions can help you to become more aware of your health conditions?

	Not Helpful 1	Reasona-bly not helpful 2	Possibly not helpful	Neutral 4	Possibly helpful 5	Basically helpful 6	Helpful 7	

This project has received funding from the European Union's Horizon 2020 research and innovation programme under the Marie Skłodowska-Curie grant agreement No 766139

			3					
Not helpful	○	○	○	○	○	○	○	Helpful

Part III

Please choose the reason why you use smart health solutions based your experiences.

14. Based on your experience, do you think smart health apps, wearable, smart home medical devices, internet based medical websites, electronic health records, e-family doctor coverage help to reduce your health risks (chronic disease management, elderly healthcare, etc.)?

	Not Helpful 1	Reasonably not helpful 2	Possibly not helpful 3	Neutral 4	Possibly helpful 5	Basically helpful 6	Helpful 7	
Not helpful	○	○	○	○	○	○	○	Helpful

15. Do you find an unstable patient-doctor Not relationship as a need to rely on smart health solutions? Do you think smart health solutions can help to improve smart health solutions?

	Not Helpful 1	Reasonably not helpful 2	Possibly not helpful 3	Neutral 4	Possibly helpful 5	Basically helpful 6	Helpful 7	
Not helpful	○	○	○	○	○	○	○	Helpful

16. Are you willing to use to share data collected from smart health solutions with hospitals or doctors?

This project has received funding from the European Union's Horizon 2020 research and innovation programme under the Marie Skłodowska-Curie grant agreement No 766139

Yes ---------------------- 1

No ---------------------- -1

17. What is your health insurance type?

I have no health insurance -------------- 1

Basic medical insurance schemes (Employee / Resident) --------- 2

Private insurance ----------------- 3

Other types of basic medical insurance schemes   ------------- 4

Free medical services for civil servants -------------- 5

I don't know the type of heath insurance I have ------------ 6

18. Do you think whether the coverage of a private insurance plan has an impact on your use of smart health solutions?

	No impact 1	With reasonable no impact 2	With possibly no impact 3	Neutral 4	With possibly an impact 5	With a reasonab le impact 6	With an impact 7	
No Impact	○	○	○	○	○	○	○	With an impact

19. Are you willing to share bio-metrics data collected from smart health devices with your insurance company (Ping An, Taikang, Zhong'an, etc)?
Yes -------------- 1
No --------------- -1

20. Do you think lack of community healthcare services is why you use smart health solutions? Are smart health solutions helpful in getting convenient health management services?

This project has received funding from the European Union's Horizon 2020 research and innovation programme under the Marie Skłodowska-Curie grant agreement No [illegible]

	Not Helpful 1	Reasonably not helpful 2	Possibly not helpful 3	Neutral 4	Possibly helpful 5	Basically helpful 6	Helpful 7	
Not helpful	○	○	○	○	○	○	○	Helpful

21. Are you willing to share the data collected from smart health solutions with community care facilities or doctors based at community care facilities?

Yes ------------ 1
No ------------- -1

Part IV

Please answer the following questions about factors affecting your use of smart health solutions, and choose the best answer based on your experience.

22. Do you think the accuracy of data collected by smart health management tools has an impact on your intention to use smart health solution?

	No impact 1	With reasonable no impact 2	With possibly no impact 3	Neutral 4	With possibly an impact 5	With a reasonable impact 6	With an impact 7	
No Impact	○	○	○	○	○	○	○	With an impact

This project has received funding from the European Union's Horizon 2020 research and innovation programme under the Marie Skłodowska-Curie grant agreement No 766139

23. Do you think privacy risk has an impact on your intention to use smart health devices?

	No impact 1	With reasonable no impact 2	With possibly no impact 3	Neutral 4	With possibly an impact 5	With a reasonable impact 6	With an impact 7	
No Impact	○	○	○	○	○	○	○	With an impact

24. Do you think the price of smart health solutions have an impact on you intention to use?

	No impact 1	With reasonable no impact 2	With possibly no impact 3	Neutral 4	With possibly an impact 5	With a reasonable impact 6	With an impact 7	
No Impact	○	○	○	○	○	○	○	With an impact

This project has received funding from the European Union's Horizon 2020 research and innovation programme under the Marie Skłodowska-Curie grant agreement No 766139

25, Do you think the brand and design of smart health solutions (look, fashion and ease to use) affect your intention of use smart health devices?

	No impact 1	With reasonable no impact 2	With possibly no impact 3	Neutral 4	With possibly an impact 5	With a reasonable impact 6	With an impact 7	
No Impact	○	○	○	○	○	○	○	With an impact

26. Have you used smart phone, apps or smart home appliances before?
Yes ------------------- 1
No -------------------- -1

27. In comparison with traditional health solutions (face-to-face communication, and keep a record with pens and pencils), are you more willing to use smart health solutions?

	1	2	3	4	5	6	7	
Willing to use traditional health management approaches	○	○	○	○	○	○	○	Willing to use smart health management tools

28. Do you think your social network (opinions of friends, family and colleagues) has an impact your attitudes towards the use smart health solutions?

This project has received funding from the European Union's Horizon 2020 research and innovation programme under the Marie Skłodowska-Curie grant agreement No 766139

	No impact 1	With reasonable no impact 2	With possibly no impact 3	Neutral 4	With possibly an impact 5	With a reasonable impact 6	With an impact 7	
No Impact	○	○	○	○	○	○	○	With an impact

Part V

Please answer questions regarding your background, and choose the best answer which fits you.

29. What one of the following options fit into your family monthly income group?

No fixed income ----------------- 0
<= 5000 RMB   ----------------- 1
5000 - 10,000 RMB (including 10,000) ------------ 2
10,000 -30,000 RMB (including 30,000) ------------- 3
30,000 - 50,000 RMB (including 50,000) ---------------- 4

30. Which one of the following options fit into your gender？

Female -------------- 0
Male ----------------- 1
I do not want to disclose this type of information. (Please leave it blank)

31.Which one of the following options fits into your education background?

Primary School ------------------- 1
Junior/Senior High School Diploma ------------ 2
Vocational Training --------------- 3
College Degree ------------- 4
Graduate School (PhD/MBA, etc) Degree --------- 5
