# Supplementary material for: Assessing Elderly User Preference for Telehealth Solutions in China: Exploratory Quantitative Study
Source: JMIR Mhealth Uhealth. 2022 Jan 12;10(1):e27272. doi: 10.2196/27272 (PMC8792775; doi:10.2196/27272)
Supplement: Multimedia Appendix 2 [file mhealth_v10i1e27272_app2.docx]

**Multimedia Appendix 2. Pearson test and Spearman rank correlation test.**

| **Table 1. Pearson Correlation Test** | | | | | | | | | | | | | |
| --- | --- | --- | --- | --- | --- | --- | --- | --- | --- | --- | --- | --- | --- |
|  | | city | age | Living situation | Regular social activity | Regular exercise | Health status | gender | education | Household income | Factor 1 | Factor 2 | Factor 3 |
| city | Pearson Correlation | 1 | .018 | -.163 | -.195 | -.113 | .181 | -.275 | .002 | -.224 | .100 | -.030 | .130 |
|  | Sig. (2-tailed) |  | .718 | .001 | .000 | .026 | .000 | .000 | .972 | .000 | .049 | .559 | .010 |
|  | N | 390 | 390 | 390 | 390 | 390 | 390 | 390 | 390 | 390 | 390 | 390 | 390 |
| age | Pearson Correlation | .018 | 1 | .069 | -.148 | .015 | .339 | .016 | -.265 | -.055 | .034 | .011 | -.022 |
|  | Sig. (2-tailed) | .718 |  | .175 | .003 | .771 | .000 | .753 | .000 | .276 | .504 | .827 | .664 |
|  | N | 390 | 390 | 390 | 390 | 390 | 390 | 390 | 390 | 390 | 390 | 390 | 390 |
| Living situation | Pearson Correlation | -.163 | .069 | 1 | .014 | .010 | -.051 | .090 | .009 | .225 | -.055 | .075 | -.062 |
|  | Sig. (2-tailed) | .001 | .175 |  | .778 | .839 | .312 | .075 | .867 | .000 | .282 | .138 | .221 |
|  | N | 390 | 390 | 390 | 390 | 390 | 390 | 390 | 390 | 390 | 390 | 390 | 390 |
| Regular social activity | Pearson Correlation | -.195 | -.148 | .014 | 1 | .329 | -.089 | .016 | .146 | .117 | -.123 | .080 | -.073 |
|  | Sig. (2-tailed) | .000 | .003 | .778 |  | .000 | .079 | .747 | .004 | .021 | .015 | .114 | .150 |
|  | N | 390 | 390 | 390 | 390 | 390 | 390 | 390 | 390 | 390 | 390 | 390 | 390 |
| Regular exercise | Pearson Correlation | -.113 | .015 | .010 | .329 | 1 | .027 | -.031 | .149 | .076 | -.110 | .101 | -.039 |
|  | Sig. (2-tailed) | .026 | .771 | .839 | .000 |  | .595 | .536 | .003 | .135 | .030 | .047 | .437 |
|  | N | 390 | 390 | 390 | 390 | 390 | 390 | 390 | 390 | 390 | 390 | 390 | 390 |
| Health status | Pearson Correlation | .181 | .339 | -.051 | -.089 | .027 | 1 | -.109 | -.143 | -.004 | .096 | -.071 | .153 |
|  | Sig. (2-tailed) | .000 | .000 | .312 | .079 | .595 |  | .032 | .005 | .931 | .059 | .161 | .003 |
|  | N | 390 | 390 | 390 | 390 | 390 | 390 | 390 | 390 | 390 | 390 | 390 | 390 |
| gender | Pearson Correlation | -.275 | .016 | .090 | .016 | -.031 | -.109 | 1 | -.025 | .064 | .014 | -.074 | -.138 |
|  | Sig. (2-tailed) | .000 | .753 | .075 | .747 | .536 | .032 |  | .617 | .207 | .785 | .145 | .006 |
|  | N | 390 | 390 | 390 | 390 | 390 | 390 | 390 | 390 | 390 | 390 | 390 | 390 |
| education | Pearson Correlation | .002 | -.265 | .009 | .146 | .149 | -.143 | -.025 | 1 | .177 | -.094 | .020 | .086 |
|  | Sig. (2-tailed) | .972 | .000 | .867 | .004 | .003 | .005 | .617 |  | .000 | .063 | .698 | .092 |
|  | N | 390 | 390 | 390 | 390 | 390 | 390 | 390 | 390 | 390 | 390 | 390 | 390 |
| Household income | Pearson Correlation | -.224 | -.055 | .225 | .117 | .076 | -.004 | .064 | .177 | 1 | -.029 | .190 | .038 |
|  | Sig. (2-tailed) | .000 | .276 | .000 | .021 | .135 | .931 | .207 | .000 |  | .570 | .000 | .452 |
|  | N | 390 | 390 | 390 | 390 | 390 | 390 | 390 | 390 | 390 | 390 | 390 | 390 |
| Factor 1 | Pearson Correlation | .100 | .034 | -.055 | -.123 | -.110 | .096 | .014 | -.094 | -.029 | 1 | .000 | .000 |
|  | Sig. (2-tailed) | .049 | .504 | .282 | .015 | .030 | .059 | .785 | .063 | .570 |  | 1.000 | 1.000 |
|  | N | 390 | 390 | 390 | 390 | 390 | 390 | 390 | 390 | 390 | 390 | 390 | 390 |
| Factor 2 | Pearson Correlation | -.030 | .011 | .075 | .080 | .101 | -.071 | -.074 | .020 | .190 | .000 | 1 | .000 |
|  | Sig. (2-tailed) | .559 | .827 | .138 | .114 | .047 | .161 | .145 | .698 | .000 | 1.000 |  | 1.000 |
|  | N | 390 | 390 | 390 | 390 | 390 | 390 | 390 | 390 | 390 | 390 | 390 | 390 |
| Factor 3 | Pearson Correlation | .130 | -.022 | -.062 | -.073 | -.039 | .153 | -.138 | .086 | .038 | .000 | .000 | 1 |
|  | Sig. (2-tailed) | .010 | .664 | .221 | .150 | .437 | .003 | .006 | .092 | .452 | 1.000 | 1.000 |  |
|  | N | 390 | 390 | 390 | 390 | 390 | 390 | 390 | 390 | 390 | 390 | 390 | 390 |

Source: Author’s illustration

| **Table 2. Spearman Rank Correlations** | | | | | | | | | | | | | | |
| --- | --- | --- | --- | --- | --- | --- | --- | --- | --- | --- | --- | --- | --- | --- |
|  | | city | age | Living situation | Regular social activity | Regular exercise | Health status | gender | education | Household income | Factor 1 | Factor 2 | Factor 3 |  |
| city | Correlation Coefficient | 1 | 0.018 | -0.206 | -0.198 | -0.112 | 0.18 | -0.276 | 0 | -0.277 | 0.093 | -0.042 | 0.135 |  |
|  | Sig. (2-tailed) | . | 0.723 | 0 | 0 | 0.027 | 0 | 0 | 0.999 | 0 | 0.068 | 0.41 | 0.008 |  |
|  | N | 390 | 390 | 390 | 390 | 390 | 390 | 390 | 390 | 390 | 390 | 390 | 390 |  |
| age | Correlation Coefficient | 0.018 | 1 | 0.067 | -0.141 | 0.02 | 0.338 | 0.011 | -0.291 | -0.06 | 0.02 | -0.005 | -0.02 |  |
|  | Sig. (2-tailed) | 0.723 | . | 0.189 | 0.005 | 0.689 | 0 | 0.825 | 0 | 0.236 | 0.701 | 0.925 | 0.693 |  |
|  | N | 390 | 390 | 390 | 390 | 390 | 390 | 390 | 390 | 390 | 390 | 390 | 390 |  |
| Living situation | Correlation Coefficient | -0.206 | 0.067 | 1 | -0.011 | -0.004 | -0.053 | 0.1 | -0.035 | 0.25 | -0.077 | 0.097 | -0.079 |  |
|  | Sig. (2-tailed) | 0 | 0.189 | . | 0.821 | 0.93 | 0.295 | 0.049 | 0.485 | 0 | 0.131 | 0.055 | 0.117 |  |
|  | N | 390 | 390 | 390 | 390 | 390 | 390 | 390 | 390 | 390 | 390 | 390 | 390 |  |
| Regular social activity | Correlation Coefficient | -0.198 | -0.141 | -0.011 | 1 | 0.329 | -0.084 | 0.016 | 0.139 | 0.126 | -0.118 | 0.054 | -0.07 |  |
|  | Sig. (2-tailed) | 0 | 0.005 | 0.821 | . | 0 | 0.098 | 0.747 | 0.006 | 0.013 | 0.02 | 0.286 | 0.165 |  |
|  | N | 390 | 390 | 390 | 390 | 390 | 390 | 390 | 390 | 390 | 390 | 390 | 390 |  |
| Regular exercise | Correlation Coefficient | -0.112 | 0.02 | -0.004 | 0.329 | 1 | 0.041 | -0.031 | 0.151 | 0.075 | -0.111 | 0.044 | -0.048 |  |
|  | Sig. (2-tailed) | 0.027 | 0.689 | 0.93 | 0 | . | 0.422 | 0.536 | 0.003 | 0.142 | 0.028 | 0.383 | 0.344 |  |
|  | N | 390 | 390 | 390 | 390 | 390 | 390 | 390 | 390 | 390 | 390 | 390 | 390 |  |
| Health status | Correlation Coefficient | 0.18 | 0.338 | -0.053 | -0.084 | 0.041 | 1 | -0.112 | -0.138 | -0.03 | 0.097 | -0.091 | 0.144 |  |
|  | Sig. (2-tailed) | 0 | 0 | 0.295 | 0.098 | 0.422 | . | 0.027 | 0.006 | 0.552 | 0.056 | 0.071 | 0.004 |  |
|  | N | 390 | 390 | 390 | 390 | 390 | 390 | 390 | 390 | 390 | 390 | 390 | 390 |  |
| gender | Correlation Coefficient | -0.276 | 0.011 | 0.1 | 0.016 | -0.031 | -0.112 | 1 | 0.008 | 0.092 | 0.014 | -0.029 | -0.147 |  |
|  | Sig. (2-tailed) | 0 | 0.825 | 0.049 | 0.747 | 0.536 | 0.027 | . | 0.873 | 0.07 | 0.783 | 0.564 | 0.004 |  |
|  | N | 390 | 390 | 390 | 390 | 390 | 390 | 390 | 390 | 390 | 390 | 390 | 390 |  |
| education | Correlation Coefficient | 0 | -0.291 | -0.035 | 0.139 | 0.151 | -0.138 | 0.008 | 1 | 0.164 | -0.086 | -0.01 | 0.059 |  |
|  | Sig. (2-tailed) | 0.999 | 0 | 0.485 | 0.006 | 0.003 | 0.006 | 0.873 | . | 0.001 | 0.088 | 0.841 | 0.245 |  |
|  | N | 390 | 390 | 390 | 390 | 390 | 390 | 390 | 390 | 390 | 390 | 390 | 390 |  |
| Household income | Correlation Coefficient | -0.277 | -0.06 | 0.25 | 0.126 | 0.075 | -0.03 | 0.092 | 0.164 | 1 | -0.009 | 0.177 | 0.023 |  |
|  | Sig. (2-tailed) | 0 | 0.236 | 0 | 0.013 | 0.142 | 0.552 | 0.07 | 0.001 | . | 0.864 | 0 | 0.653 |  |
|  | N | 390 | 390 | 390 | 390 | 390 | 390 | 390 | 390 | 390 | 390 | 390 | 390 |  |
| Factor 1 | Correlation Coefficient | 0.093 | 0.02 | -0.077 | -0.118 | -0.111 | 0.097 | 0.014 | -0.086 | -0.009 | 1 | -0.068 | 0.036 |  |
|  | Sig. (2-tailed) | 0.068 | 0.701 | 0.131 | 0.02 | 0.028 | 0.056 | 0.783 | 0.088 | 0.864 | . | 0.183 | 0.48 |  |
|  | N | 390 | 390 | 390 | 390 | 390 | 390 | 390 | 390 | 390 | 390 | 390 | 390 |  |
| Factor 2 | Correlation Coefficient | -0.042 | -0.005 | 0.097 | 0.054 | 0.044 | -0.091 | -0.029 | -0.01 | 0.177 | -0.068 | 1 | -0.019 |  |
|  | Sig. (2-tailed) | 0.41 | 0.925 | 0.055 | 0.286 | 0.383 | 0.071 | 0.564 | 0.841 | 0 | 0.183 | . | 0.713 |  |
|  | N | 390 | 390 | 390 | 390 | 390 | 390 | 390 | 390 | 390 | 390 | 390 | 390 |  |
| Factor 3 | Correlation Coefficient | 0.135 | -0.02 | -0.079 | -0.07 | -0.048 | 0.144 | -0.147 | 0.059 | 0.023 | 0.036 | -0.019 | 1 |  |
|  | Sig. (2-tailed) | 0.008 | 0.693 | 0.117 | 0.165 | 0.344 | 0.004 | 0.004 | 0.245 | 0.653 | 0.48 | 0.713 | . |  |
|  | N | 390 | 390 | 390 | 390 | 390 | 390 | 390 | 390 | 390 | 390 | 390 | 390 |  |

Source: Author’s illustration

Table 3. VIF Score

| Variable | VIF | 1/VIF |
| --- | --- | --- |
|  |  |  |
| Health status | 1.24 | 0.806738 |
| city | 1.24 | 0.807404 |
| age | 1.24 | 0.808972 |
| Social activity | 1.21 | 0.829270 |
| income | 1.19 | 0.841438 |
| Regular exercise | 1.17 | 0.854727 |
| education | 1.16 | 0.859024 |
| gender | 1.12 | 0.895233 |
| Living situation | 1.09 | 0.918591 |
| Factor 3 | 1.07 | 0.933362 |
| Factor 2 | 1.07 | 0.935581 |
| Factor 1 | 1.04 | 0.959257 |
|  |  |  |
| Mean VIF | 1.15 |  |

Source: Author’s illustration
